# Salivary Biosensing Opportunities for Predicting Cognitive and Physical Human Performance

**DOI:** 10.3390/bios15070418

**Published:** 2025-07-01

**Authors:** Sara Anne Goring, Evan D. Gray, Eric L. Miller, Tad T. Brunyé

**Affiliations:** 1 Center for Applied Brain and Cognitive Sciences, Tufts University, Medford, MA 02155, USA; sara.goring@tufts.edu (S.A.G.); evan.gray@tufts.edu (E.D.G.); eric.miller@tufts.edu (E.L.M.); 2 Department of Electrical and Computer Engineering, Tufts University, Medford, MA 02155, USA; 3 U.S. Army DEVCOM Soldier Center, Natick, MA 01760, USA

**Keywords:** biosensor, cortisol, stress, hormones, metabolites, enzymes, physical performance, cognitive performance

## Abstract

Advancements in biosensing technologies have introduced opportunities for non-invasive, real-time monitoring of salivary biomarkers, enabling progress in fields ranging from personalized medicine to public health. Identifying and prioritizing the most critical analytes to measure in saliva is essential for estimating physiological status and forecasting performance in applied contexts. This study examined the value of 12 salivary analytes, including hormones, metabolites, and enzymes, for predicting cognitive and physical performance outcomes in military personnel (N = 115) engaged in stressful laboratory and field tasks. We calculated a series of features to quantify time-series analyte data and applied multiple regression techniques, including Elastic Net, Partial Least Squares, and Random Forest regression, to evaluate their predictive utility for five outcomes of interest: the ability to move, shoot, communicate, navigate, and sustain performance under stress. Predictive performance was poor across all models, with R-squared values near zero and limited evidence that salivary analytes provided stable or meaningful performance predictions. While certain features (e.g., post-peak slopes and variance metrics) appeared more frequently than others, no individual analyte emerged as a reliable predictor. These results suggest that salivary biomarkers alone are unlikely to provide robust insights into cognitive and physical performance outcomes. Future research may benefit from combining salivary and other biosensor data with contextual variables to improve predictive accuracy in real-world settings.

## 1. Introduction

Advances in salivary biosensing have positioned saliva as a powerful, non-invasive medium for real-time biomarker detection, with applications in personalized health monitoring, sports performance, and clinical diagnostics. Saliva is an increasingly informative biofluid due to the development of biosensors capable of detecting and quantifying a diverse array of analytes, including hormones (e.g., cortisol, testosterone), metabolites (e.g., lactate, creatinine), enzymes (e.g., alpha amylase), electrolytes (e.g., sodium, potassium), and antibodies (e.g., immunoglobulin). For example, electrochemical and optical sensors can measure concentrations of cortisol to understand acute and chronic stress levels and well-being [1,2,3], lactate to understand metabolic status [4,5,6], and antibody titers to understand immune response [7,8]. These are potentially transformative developments for tracking health and well-being.

While salivary analytes may carry value for understanding physiological states, less is known about whether they may also predict human performance on challenging cognitive and physical tasks. Several occupations, including military personnel, industrial workers, and first responders, rely upon employees maintaining their performance as they become stressed, fatigued, thermally strained, calorie-deprived, dehydrated, or otherwise experience disrupted physiological homeostasis [9,10,11,12,13,14,15,16]. In high-stakes environments, these suboptimal states may negatively affect performance on a range of cognitive and physical functions. For example, strenuous bouts of physical exertion can cause physical fatigue [17], modulate lactate concentrations in saliva and blood [18,19,20], and adversely affect performance on simple reaction time and executive function tasks [21,22,23]. Similarly, exposure to unpredictable, threatening, novel, or uncontrollable circumstances can cause acute stress responses [24,25,26,27], modulate cortisol concentrations in saliva [28,29,30], and adversely affect performance on memory, executive function, and decision-making tasks [31,32,33,34,35,36].

There are several different salivary biosensor formats. Some involve collecting and analyzing passive drool at the point of need, for example, with a handheld sensor [37]. Other biosensors are worn in the oral cavity, for example, affixed to the surface of a tooth [38] or integrated into a mouthguard [39,40,41]. The cost and complexity of biosensor development and use increase in proportion to the number of analytes. Multiplexed biosensors are preferred, but scaling from 2 to *n* targeted analytes becomes increasingly expensive and complex to fabricate and requires advanced signal processing and complex data interpretation [42,43,44]. Therefore, identifying and prioritizing the most critical analytes—those that are both reliably predictive and highly informative for key performance outcomes—is essential to maximize the impact and efficiency of biosensing technology [45,46]. By focusing on these high-value analytes, biosensor design can be streamlined, reducing costs while ensuring that the resulting data are applicable and actionable for decision-making.

To inform the prioritization of targeted analytes for biosensing in the context of military training and operations, the present study examined the relative utility of 12 salivary analytes for predicting cognitive and physical performance outcomes. A large sample of military personnel participated in stressful research events while performing a wide range of occupational tasks. We asked whether performance on these tasks is predicted by time-series changes in salivary analyte concentrations. Below, we detail the measured analytes and performance outcomes.

### 1.1. Measured Analytes

Analytes were measured using passive drool or salivette techniques. To collect data on a relatively comprehensive set of salivary analytes that have previously demonstrated value for understanding ongoing physiological states, we sampled a range of hormones, metabolites, enzymes, electrolytes, and antibodies. To assess hormones, we measured concentrations of cortisol, estradiol, testosterone, and dehydroepiandrosterone (DHEA). To assess metabolites, we measured concentrations of creatinine, urocanic acid, lactate, arginine, carnosine, carnitine, and histidine. Finally, we measured concentrations of the enzyme alpha amylase. As detailed in Table 1, each of these analytes has previously been implicated in one or more aspects of physiological functioning relevant to cognitive or physical performance.

The selection of the 12 salivary analytes was motivated by their mechanistic linkage to physiological systems that plausibly influence cognitive and physical performance. Hormones such as cortisol, testosterone, and DHEA are closely tied to the hypothalamic–pituitary–adrenal (HPA) axis and hypothalamic–pituitary–gonadal (HPG) axis, which regulate stress reactivity, energy balance, motivation, and anabolic processes, all of which can modulate readiness and resilience under demanding conditions [47,48,49,52,53,54,55,56,57]. Metabolites like lactate, carnitine, and carnosine are implicated in muscle metabolism and recovery, with links to physiological fatigue and endurance capacity [64,65,66,67,68,71,72,73,74,75]. Similarly, arginine and histidine play roles in nitric oxide production and immune modulation, linking systemic inflammation to physical and cognitive function [69,70,76,77]. Creatinine reflects muscle mass and kidney function, indirectly indexing hydration and exertion status [58,59,60]. Alpha amylase is a well-validated marker of sympathetic nervous system activation and has been associated with acute cognitive arousal and performance variability in high-stress contexts [78,79,80,81]. Together, the analytes were selected to reflect key systems (i.e., endocrine, metabolic, immune, and autonomic) that are mechanistically linked to cognitive and physical performance.

### 1.2. Measured Outcomes

Participants engaged in a series of tasks that demanded moving, shooting, communicating, navigating, and sustaining readiness [10,82]. In military contexts, the ability to move effectively as a member of a team is essential for avoiding enemy targeting, moving between cover and concealment options, and assuming effective offensive positions. Shooting is a critical element of infantry function, involving the employment of a weapon (typically a rifle) to engage stationary or dynamic targets at close or far ranges. Communicating, typically by voice, is essential for conveying simple or complex ideas to facilitate team-coordinated performance. Navigating involves moving effectively between distal objectives while relying upon environmental memory, situation awareness, and supporting devices (e.g., compass, geographical positioning device, map). Finally, military personnel are expected to sustain physiological and neuromuscular readiness throughout the duration of strenuous events, allowing them to maintain effective movement and visuomotor and mental function. Our analyses tested whether features derived from time series data of several salivary analytes would predict performance variation on each of these five outcomes.

### 1.3. Hypotheses

Salivary analytes are widely regarded as proxies for transient physiological states such as stress, dehydration, and fatigue. However, prior research highlights several sources of variability and unreliability in associating salivary analyte concentrations with underlying physiological states. These include inter- and intra-individual variability, circadian rhythms, differences in salivary flow rate, and environmental or behavioral factors that influence salivary composition [83,84,85]. Similarly, while physiological states influence cognitive and physical performance, this connection is often indirect, context-dependent, and influenced by numerous interacting factors [10,86,87,88,89]. Given those challenges and the second-order nature of the relationship between salivary analytes, physiological states, and performance outcomes, we expect that time-series changes in salivary analyte concentrations will have limited predictive utility for our five performance outcomes of interest.

This hypothesis is grounded in two primary extant findings. First, cognitive performance reflects complex interactions among psychological, physiological, and environmental factors, and salivary analytes have shown inconsistent relationships with specific cognitive outcomes [28,86]. For example, biomarkers of acute stress often fail to predict task performance under high-stakes conditions due to variability in individual stress resilience and task complexity [32,90]. Second, physical performance depends on physiological readiness, including neuromuscular activation, hydration, and energy metabolism. While analytes such as lactate can provide a snapshot of metabolic activity, they often fail to directly correlate with task-specific physical outcomes due to confounding variables like training status or task context [67,87,91].

## 2. Materials and Methods

A total of 115 military personnel participated in at least one of six study events over the course of 4–6 weeks. Written informed consent was obtained from all participants in accordance with ethical approvals (protocol numbers detailed in Table 2). The study events are detailed in Table 2. For more details on each study protocol, please refer to the referenced articles.

All study events were intended to induce mental and physical stress and provide opportunities to measure performance on individual and/or collective (i.e., team-level) tasks. Saliva samples were collected from participants at multiple time points as they participated in a study event (Table 2); note that the first sample (i.e., time point 1) was always collected immediately prior to the stressor. Biomarker concentrations of each analyte were measured in triplicate using enzyme-linked immunosorbent assays (ELISAs). Immediately after collection, saliva samples were stored for up to 1 day at −20 °C and then at −80 °C for up to 3 months prior to assays. For assays, the samples were thawed on ice, aliquoted, centrifuged, and analyzed in duplicate or triplicate using Salimetrics (State College, PA, USA), Eagle Biosciences (Amherst, NH, USA), and/or Thermo Fisher Scientific (Waltham, MA, USA) assays (depending on the protocol). Biomarker data were then standardized, and outliers exceeding ± 2.5 *SD* were removed and mean-imputed. Data were then log_10_ transformed to conform with traditional biomarker analytical practices.

### 2.1. Software

Initial data cleaning, regression diagnostics, and imputation were completed in R Studio (R Version 4.4.1). Correlation analysis was performed using the olsrr package (Version 0.6.0). Imputation was performed using Multivariate Imputation by Chained Equations with the Mice package (Version 3.16.0). The remaining steps, including feature calculations, statistical modeling, and data visualization, were conducted using Python, via Jupyter Notebooks (Python Version 3.8.8) [97]. Data manipulation and feature calculation were performed using the SciPy (Version 1.6.2) [98], NumPy (Version 1.22.3) [99], and Pandas (Version 1.3.5) [100] packages. Modeling was performed using the Scikit-learn package (Version 0.24.1) [101]. Visualization was performed using the Matplotlib (Version 3.3.4) [102] and Seaborn (Version 0.11.1) [103] packages.

### 2.2. Feature Calculations

**Figure 1:**
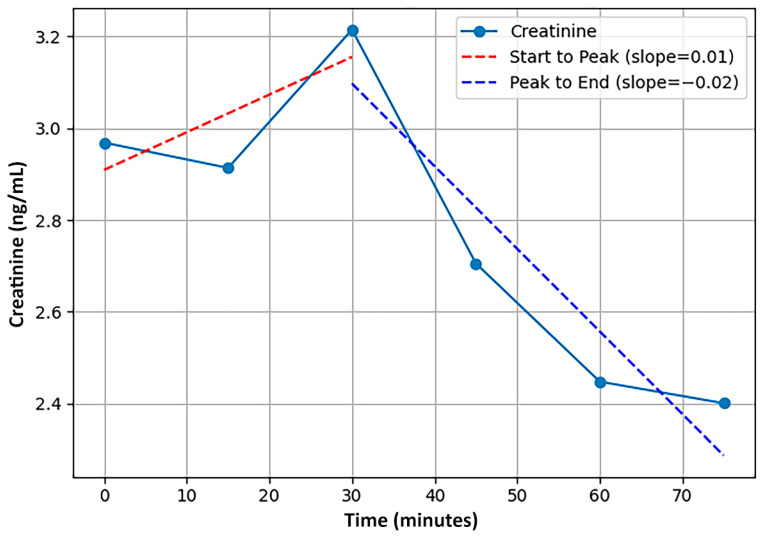
Example participant time series (in minutes) data points reflecting the calculation of slope-based features: Slope (start to peak) and Slope (peak to end).

When evaluating the concentration of analytes in saliva samples across variable time series, group-level metrics averaged across a sample fail to accurately capture patterns that occur at the individual level [104,105]. Instead, we calculated a series of six individual-level features that quantified the temporal dynamics of the time series (detailed in Table 3) and have been examined in previous biomarker and pharmacokinetic research [106,107,108,109,110]. See Figure 1 for a visualization of two of the features that utilized the slope between time points in a series for a single participant. Note that two features that were initially calculated were eliminated due to being redundant with other features, a third was discarded for lack of sufficient samples for accurate calculation, and a fourth was not used for lack of interpretability. In greater detail, first, the area under the curve (AUC) feature was highly correlated with cMEAN and was incompatible with the varying time-series lengths; cMEAN was retained to reduce feature redundancy and multicollinearity. Second, cDIFF (the difference between cMAX and cMIN) was highly correlated with variance, and the latter was retained. Third, an entropy feature was not employed, given the low number of points in some time series observations. Finally, a feature representing the overall slope across time points was discarded due to a lack of interpretability.

Calculated feature values were averaged across study events, so each participant had a standardized value of each feature for each of the 12 measured analytes. Because baseline (i.e., first time point) analyte concentrations varied dramatically across participants, all features were calculated using baseline-corrected data. Specifically, for each participant, the first data point (baseline) was subtracted from all subsequent data points, ensuring that each participant’s data were normalized to their own baseline.

### 2.3. Performance Outcome Calculations

Based on quantitative and qualitative data from each study event’s tasks, composite performance outcomes were calculated as detailed in our previous report [10]. Briefly, 50 outcome metrics were derived from the study events and categorized into five outcome domains of Move, Shoot, Communicate, Navigate, and Sustain based on an expert consensus panel. The outcome metrics were then standardized and aggregated into a single standardized composite measure for each of the five outcomes and each participant. Because participants did not necessarily engage in all of the events, the total number of observations within each outcome domain varied: Move (87), Shoot (115), Communicate (84), Navigate (83), Sustain (110).

### 2.4. Regression Diagnostics

Highly correlated features (*r* ≥ 0.70) were evaluated for multicollinearity with variance inflation factors (VIFs). Features with the highest VIFs over 5 were removed individually until all VIF values fell below that threshold [111]. Variables and observations with missing data exceeding 50% were discarded. Finally, data imputation was conducted on any remaining features with missing data using multiple-imputation-by-chained equations (MICE) techniques [112]. Note that outcome data were not included in the MICE technique to avoid contamination. See Table 4 for sample size, feature reduction, and details of imputed values.

### 2.5. Outliers

After performing filtering and correlation analysis, outliers were identified for each of the features. Outliers were determined as data values exceeding ± 1.5 Interquartile Range (IQR). Across each feature within each domain, there was an average of four outlying data values, with each individual having around two outlying data values per domain. There was no discernible pattern regarding outliers at the feature or individual level. Most features and individuals had at least a few outlying values. As a result, these features and individuals with outliers were noted but maintained in the dataset for the modeling and analysis procedures. They were also maintained to avoid a second round of data imputation. See Table 5 for the distribution of outliers by feature and participant.

### 2.6. Modeling and Analysis

Due to the number of features and the potential for complex interrelations among them, multiple analytical approaches were employed to explore the associations in the data. Overall, three regression methods were used for each of the five different outcome domains. Each technique used was intended to offer a unique perspective not captured by the other analytic methods. As detailed below, the general inability of all three to find meaningful relations between saliva analytes and any of the performance measures lends support to our governing hypothesis. The distinct viewpoint each technique contributes is detailed below, along with a description of the modeling procedures. For each of the 15 cases, the associated hyperparameters were optimized using a 90/10 train-test split with 10-fold cross-validation on the training set. To examine stability across samples, each model was evaluated using 100 different 80/20 training–validation splits of the training set [113,114].

#### 2.6.1. Elastic Net

Elastic Net regression with grid search-tuned hyperparameters was explored as a way to improve performance compared to linear regression. This technique was selected for its ability to handle high-dimensional datasets with correlated predictors, enabling interpretable feature selection while controlling for multicollinearity, which is common in biomarker time-series data. Combining the advantages of both Lasso and Ridge regression techniques, Elastic Net selects only the most important features, while removing or shrinking the weights of features with less predictive power [105]. Hyperparameters were selected using a grid search with 10-fold cross-validation on the training set. For the Elastic Net model, the tuning approach was clearly defined, focusing on the regularization parameters alpha and l1_ratio. The grid search was performed using *Scikit-learn’s ElasticNetCV* implementation. In this implementation, the smallest α($\alpha_{max}$) that sets all coefficients to zero is calculated, and a logarithmic sequence of 100 alpha values between $0.001\;*\;\alpha_{max}$ and $\alpha_{max}$ is tested. Additionally, nine L1 mixing parameter values distributed evenly between 0.1 and 0.9 were tested in this grid search. Mean squared error (MSE) was used as the optimization criterion [115].

#### 2.6.2. Partial Least Squares

Partial Least Squares regression (PLS-R) is a machine-learning technique that combines characteristics from principal components analysis and multiple regression analysis [108]. PLS-R exhibits strength in modeling datasets with many highly collinear predictors and few observations, making it well-suited for extracting latent biomarker–performance relationships from complex physiological data. The common covariance between the predictors and the outcome is decomposed into components, and these latent vectors are used to conduct the regression analysis step. PLS-R techniques were employed with variable importance in projection (VIP) [109,110] feature selection to identify uncorrelated latent structures among highly correlated features. For each model, features with VIP scores below 0.8 were iteratively removed. Models were tested with latent components ranging from 1 to 10, and the optimal number of components was determined based on validation MSE.

#### 2.6.3. Random Forest

Random Forest regression was used to explore potential nonlinear relationships and feature importance within those relationships. This technique affords the exploration of potential nonlinear interactions and feature combinations, offering robustness to overfitting and flexibility in modeling complex biological relationships that may not follow linear assumptions. The Random Forest approach is an ensemble learning method that attempts to group classes of features together via the output of multiple decision trees [111]. In this implementation, 100 estimators were fitted using an MSE split quality criterion. For the Random Forest model, n_estimators (e.g., 100, 200, 500), max_depth (e.g., 5, 10, 20, None), min_samples_split (e.g., 2, 5, 10), and min_samples_leaf (e.g., 1, 2, 4) were tuned over a range of commonly used values. RMSE (Root Mean Squared Error) was used as the optimization criterion to select the best hyperparameter combination. To prevent data leakage, all hyperparameter tuning and cross-validation were performed strictly on the training set. While full nested cross-validation was not used, the separation between training and test sets ensured that model selection remained unbiased by test set performance.

## 3. Results

### 3.1. Participant Demographics

Demographic details regarding the 115 participants are included in Table 6. All participants were male, and in terms of ethnicity, the sample included 86 white non-Hispanic, 16 Hispanic, six black non-Hispanic, three Asian, two Pacific islander, one American Indian/Alaskan native, and one other non-specified individuals.

### 3.2. Elastic Net Results

The models built with the Elastic Net approach tended to perform the worst overall among the modeling techniques considered. The primary motivation behind using Elastic Net was to identify features that exhibit a linear relationship with the outcome while penalizing those that do not. In practice, a limited number of features were selected across most models, contributing to the overall poor performance as measured by near-zero (0) *R^2^* scores and near-one (1) root mean squared error (RMSE). See Table 7 for overall model results.

**Figure 2:**
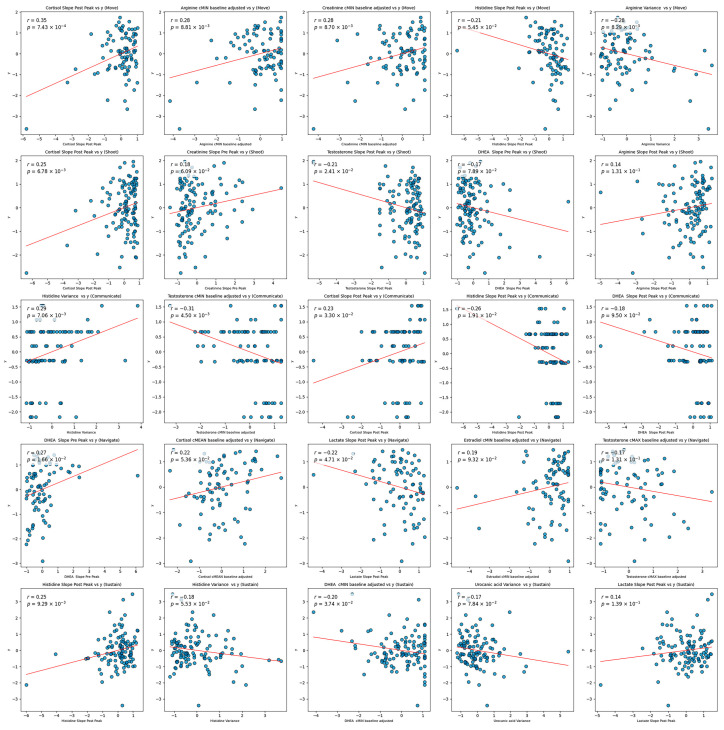
Scatter plots displaying the relationship between a single z-scored feature and the z-scored target with a simple linear regression line fit to each (with corresponding r values and *p*-values). Note. The features apparent in these plots were the top five most highly selected features, on average, across models for each domain.

The sparsity suggests that many features had weak or non-linear relationships with the target variable, which Elastic Net inherently penalizes. The models were optimized over MSE, a loss function that penalizes large errors more heavily. Models with a higher mixture parameter (leaning more towards Lasso regression) performed marginally better than those with a lower mixture parameter (closer to Ridge regression). This preference for Lasso indicates that the model favored greater sparsity by focusing on a smaller subset of features. This subset, however, may have excluded potentially important features with weaker linear associations, ultimately limiting predictive power. The fact that a naive model that predicts the target based solely on the mean value of the training set outperformed these complex, sparse models underscores the lack of strong, stable relationships between individual features and the target. Sparse models were not likely to have much success due to the seemingly random relationships between individual features and the target. As shown in the scatter plots in Figure 2, there appears to be weak, if any, relationships between the most highly selected features and the target. This behavior is likely a reason for the lack of stability in the models. Certain samples in the testing set may be representative of others in the training set, but this behavior is not consistent and therefore prevents general conclusions. See Table 7 for overall model results.

### 3.3. Partial Least Squares Results

The models built with Partial Least Squares regression tended to perform poorly, although slightly better than Elastic Net. This approach was intended to handle the case of multicollinearity inherent in building out features from the same time series data points. The features selected using the VIP approach displayed a pattern where a small number of features were chosen frequently, while the majority were selected less often. See Table 7 for overall model results.

The Partial Least Squares regression models demonstrated a similar resistance to generalization across iterations. By design, the average of the squared VIP scores is 1 [116]. A threshold of 0.8 was set for feature selection to ensure the models retained some level of predictive capacity by guaranteeing that each iteration included at least one nonzero coefficient. Despite this, the models were generally sparse, as reflected by the sharp decline in the frequency of selected features. This pattern suggests that while certain features were consistently selected, they likely contributed to the latent variables that have high covariance with the target. Partial Least Squares regression works by projecting both the predictor and response variables into a lower-dimensional space, where it identifies latent variables that maximize the covariance between the two. PLS therefore captures the most predictive dimensions in the data, even in the presence of high collinearity. In this context, the consistently selected features are likely those that load heavily onto the latent variables with the strongest association with the target. This suggests that across different splits of the data, these features carry information most relevant to the response, even if the overall model fit is low. The relatively sparse models and the low predictive performance indicate that, despite identifying some features with strong covariance to the target, the extracted latent variables may not capture enough meaningful variance in the response to enable robust predictions.

### 3.4. Random Forest Results

The Random Forest models tended to perform on par with the PLS models. This approach was used to examine the usage of a nonlinear model compared to linear models. Perhaps one of the most notable differences between the Random Forest models and the linear models is the distribution of the frequencies of selected features. In the Random Forest models, features tended to be selected across iterations with less consistency. In other words, different splits in the data resulted in highly different models with reference to the features selected. In the linear models, most features are selected infrequently, with few features selected frequently. Compared to the Random Forest models, certain features were selected in the linear models in most, if not all, of the iterations, while others appeared sporadically. See Table 7 for overall model results.

Despite the models having similar overall poor performance, the behavior of the Random Forest models was somewhat different from that of the linear models. In these models, the frequency of features selected is distributed relatively normally. This might suggest the lack of highly selected features that can be described in a linear model. Random Forests rely on multiple decision trees that each capture different subsets of features and complex feature interactions. In contrast, linear models directly attribute importance to features based on linear relationships, making highly predictive features stand out more clearly and be selected more consistently. There might be interactions between features due to the inherently interconnected nature of the human body. As a result, the relationship between saliva biomarkers and performance may be complex and nonlinear. A slight improvement over the linear models is most apparent in the Move domain, where 39 of the models explained a non-zero level of variance compared to only 17 of the PLS models. Still, this represents fewer than 50% of the models created with different sample splits and demonstrates a strong resistance to generalization.

### 3.5. Overview

The performance of these models was poor, with high resistance to generalization. Across each modeling method, fewer than half of the 100 models tended to explain any level of variance in the test set. As a result, *R*^2^ values tended to be arbitrarily low. Directional predictive accuracy (i.e., the correct sign of predictions) was, on average, approximately 50%, indicating a near-random level of performance. While this may not necessarily indicate the poor performance of a predictive model, the average root mean squared error (RMSE) across each set of models in each domain was near 1. This relatively high RMSE, coupled with the low level of directional predictive accuracy, indicates that not only do the models fail to predict a true positive or true negative outcome, but those predictions tend to be far from the true value, whether positive or negative. Notably, occasional low RMSE or high *R*^2^ values were likely artifacts of sampling rather than genuine generalization. No single feature or analyte was selected widely across models and outcome domains. Features related to the post-peak and pre-peak slopes of biomarker concentration frequently appeared and may be informative about the body’s handling of such analytes.

The poor overall performance of each model suggests that biomarkers alone may not be optimal predictors of performance; unfortunately, our current data do not allow us to test whether additional and qualitatively different predictors might prove more valuable. The linear model feature selection methods tended to select the same features with similar frequency for each of the domains. Certain features, such as Cortisol post peak slope and Arginine variance in Move, Cortisol post peak slope in Shoot, Histidine variance and Testosterone cMIN in Navigate, and Histidine post peak slope in Sustain, appear consistently across both linear model selection methods. Although these features are frequently selected in models built from different subsets of the data, their inclusion does not lead to the creation of adequate models. The failure of the models to generalize speaks to the overall performance of using such biomarker features in a predictive model of human cognitive and physical performance outcomes.

One possible reason for the difficulty in achieving strong predictive performance may be the inherent variance of the target variable. High variability in the response can limit the model’s ability to capture reliable patterns, particularly when working with a limited set of predictive features and observations. Although the low model fit precludes definitive conclusions about the effects of selected features, the features frequently selected across iterations may still warrant further investigation.

To understand poor model performance, the top five features from each domain were *z*-score-normalized and plotted against the respective standardized target (see Figure 2). There does not appear to be a notable linear relationship between any of these features and the target. High target values appear almost equally often for low and high feature values. This would suggest a potentially high impact of noise on the data or the lack of an effective linear model. Additionally, the regression line appears to be heavily impacted by outliers and leverage data values. Such behavior might explain the slope in the plots.

**Figure 3:**
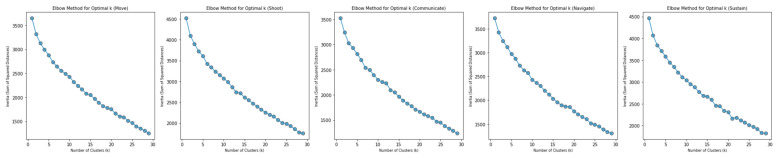
Elbow plots displaying the relationship between the number of clusters, k, and the sum of squared distances between each point and its closest centroid.

Cluster analysis (using K-Means) was performed with the intention of identifying potential groupings in the data. Elbow plots of k versus inertia across each domain were relatively linear, which suggests an absence of meaningful clusters. See Figure 3 for elbow plots. Note that the value 30 was used as an upper bound for k to avoid the excessive creation of single-member clusters.

## 4. Discussion

The goal of the current study was to examine the predictive value of salivary analytes for cognitive and physical performance outcomes in military personnel under stressful conditions. We hypothesized that time-series changes in 12 salivary analytes would demonstrate limited predictive value for these outcomes, given the known complexities in linking salivary biomarkers to transient physiological states and human performance. Our findings aligned with this hypothesis. Across a broad range of modeling techniques (Elastic Net, PLS, and Random Forest), predictive performance was consistently poor. Indeed, the models exhibited near-zero R-squared values, indicating a minimal ability to explain variance in performance outcomes, and the predictive accuracy of directional trends remained near chance levels, with RMSE approaching one. Notably, none of the analytes emerged as a reliable or consistent predictor across domains. Although certain features, such as post-peak slopes and variance metrics, appeared frequently in selected models, their inclusion failed to improve predictive performance beyond chance levels. Overall, these results suggest that biomarkers alone may not provide robust insight into cognitive and physical performance outcomes in military contexts.

### 4.1. Theoretical and Practical Implications

Theoretically, the present results provide some insights into the complexity of linking physiological states directly to real-world performance outcomes. Previous studies have suggested that salivary analytes, particularly those associated with stress (e.g., cortisol, alpha-amylase), may track meaningful changes in cognitive and physical readiness in relatively uncontrolled contexts. However, our results reinforce the notion that such relationships are highly variable and/or weak, and also likely dependent on numerous contextual factors. This underscores the challenge of isolating reliable salivary biomarkers that reflect underlying physiological processes with sufficient stability to predict real-world performance outcomes. The weak predictive associations identified here suggest that physiological markers alone may be inadequate without complementary information about task demands, environmental context, or individual differences.

Furthermore, it is possible that salivary analytes alone may not be sufficient to reliably classify the mental or physical states that directly relate to performance. Multiplexed sensors that detect several analytes may prove more valuable than individual analytes. Given the added complexity and cost associated with multiplexed biosensors, trade-offs must be considered between complexity, cost, and effectiveness.

From an applied perspective, the present results carry implications for the development and deployment of biosensing technologies. The inability to identify predictive biomarkers highlights the need for multimodal approaches that combine salivary biosensing with complementary physiological, behavioral, and contextual measures. For example, integrating heart rate variability, electrodermal activity, sleep duration, or movement data alongside salivary cortisol may help disambiguate the precise internal states driving performance (e.g., fatigue versus psychological stress). Such integration can improve model robustness and classification accuracy in real-world settings [117,118,119,120,121]. Multimodal data fusion can leverage temporal dynamics and interdependencies between modalities to improve the prediction of outcomes such as cognitive workload, vigilance, or readiness. Thus, algorithms designed to predict performance may benefit from incorporating rich data streams such as task performance, voice acoustics, calorie intake, thermal burden, load carriage, and metabolic status, supporting a systems-level approach to biosensing that aligns with operational demands and increases the ecological validity of predictions.

### 4.2. Strengths and Limitations

Key strengths of this study include its comprehensive examination of salivary analytes across multiple outcome domains in a large sample of military personnel. The use of multiple modeling approaches strengthened the robustness of our conclusions by revealing consistent patterns of weak predictive value. Additionally, our feature engineering process effectively explored potential interpretable time-series patterns in analyte data that could otherwise have been overlooked. Each defined feature provided meaningful insights into the temporal behavior of the analytes.

While the study incorporated rigorous data cleaning and imputation procedures, it is important to recognize that variability in performance outcomes and salivary analytes is an inherent characteristic of real-world applications. This variability reflects the complexity of deploying such measures outside tightly controlled laboratory settings and stems from fluctuations in individual performance across tasks or from extrinsic factors influencing saliva composition (e.g., hydration status, circadian variation). These sources of variability underscore the challenge and opportunity of identifying salivary biomarkers that remain robust and predictive under naturalistic conditions. Similarly, while our sampling strategy used discrete time points, future work may benefit from more temporally resolved sampling to better characterize dynamic biomarker fluctuations.

Future research may benefit from exploring deep learning architectures, such as long short-term memory (LSTM) networks or Transformer-based models. These methods have demonstrated success in modeling nonlinear temporal dependencies in physiological time-series data and may be able to uncover patterns not captured by regression-based approaches. However, their application also introduces increased model complexity, reduced interpretability, and a need for substantially larger datasets to avoid overfitting. Given the relatively small sample size and modest time-series resolution in the present study, we prioritized models that emphasized interpretability and transparency. Future work with larger datasets and denser sampling may be better suited to leveraging the potential advantages of deep learning methods for understanding complex biomarker–performance relationships.

## 5. Conclusions

Overall, we provide evidence that salivary analytes have limited predictive utility for cognitive and physical performance outcomes under stress. The findings emphasize the need for integrative approaches that combine biosensor data with contextual information when attempting to predict human performance in operational environments. Biosensing alone may not be sufficiently robust to reliably predict performance; with real-world contexts and tasks, prediction is a critical step for translating status into actionable guidance. Future research should explore hybrid models that integrate physiological, behavioral, and environmental data to better understand performance variability in high-stakes contexts.

## Figures and Tables

**Table 1:** The 12 analytes measured, their biomarker class, and their relevance to human physiology and performance.

Class	Analyte	Relevance
Hormone	Cortisol	Stress, Fatigue, Recovery, Readiness [47,48,49]
Hormone	Estradiol	Bone and Muscle Health, Menstrual Cycle Phases [50,51]
Hormone	Testosterone	Muscle Strength, Energy, Motivation, Stress [52,53,54]
Hormone	DHEA	Stress Resilience, Immune Function [55,56,57]
Metabolite	Creatinine	Muscle Health, Kidney Function [58,59,60]
Metabolite	Urocanic Acid	Skin Health, Immune Function, Histidine Metabolism [61,62,63]
Metabolite	Lactate	Muscle Fatigue, Exercise Intensity, Oxygen Demand [64,65,66,67,68]
Metabolite	Arginine	Immune Function, Wound Healing, Nitric Oxide Production [69,70]
Metabolite	Carnosine	Muscle Recovery, Oxidative Stress, Acid-Base Balance [71,72]
Metabolite	Carnitine	Energy Metabolism, Muscle Recovery, Endurance Capacity [73,74,75]
Metabolite	Histidine	Immune Function, Tissue Repair, Antioxidant Availability [76,77]
Enzyme	Alpha Amylase	Stress, Sympathetic Nervous System Activation, Alertness and Readiness [78,79,80,81]

**Table 2:** The study events, IRB-approved study protocol numbers, study settings, number of bio sampling time points (and time interval between samples, in minutes), and salivary biomarkers collected.

Study Event, Protocol Number, Reference(s)	Study Setting	Sample Count (Time Interval)	Biomarkers Collected (Units for First Mention)
Cognitive Prediction Study; Protocol #18-007; [89,92]	Laboratory	5 (20)	Alpha Amylase (U/mL), Cortisol (µg/dL), Estradiol (ng/mL), Testosterone (ng/mL), DHEA (ng/mL), Creatinine (ng/mL), Urocanic Acid (ng/mL), Lactate (ng/mL), Arginine (ng/mL), Carnosine (ng/mL), Histidine (ng/mL).
Physical Prediction Study; Protocol #17-002; [93]	Laboratory	5 (20)	Cortisol, DHEA, Creatinine, Urocanic Acid, Lactate, Arginine, Carnitine (µmol/L), Histidine, Testosterone.
Small-unit Performance Analytics (SUPRA); Protocols 20-001 & 18-003; [94]	Field	2 (100)	Cortisol, DHEA, Creatinine, Urocanic Acid, Lactate, Arginine, Carnitine, Testosterone, Estradiol.
Tactical Stress Marksmanship Assessment (TSMA); Protocol 20-008; [95,96]	Field	6 (15)	Cortisol, DHEA, Creatinine, Urocanic Acid, Lactate, Arginine, Carnitine, Histidine, Testosterone, Estradiol

**Table 3:** The six calculated features characterizing the biomarker time series data, and a description of each.

Feature	Description
cMAX	Maximum effective concentration in the time series.
cMIN	Minimum effective concentration in the time series.
cMEAN	Mean analyte concentration in the time series.
Slope (start to peak)	Linear slope from the first time point in series to the time point of cMAX.
Slope (peak to end)	Linear slope from time point of cMAX to the last time point in series.
Variance	Sample variance of all the time points in the time series.

**Table 4:** Relevant numbers related to sample size, feature size, and imputation.

Domain	Initial Sample Size	Final Sample Size	Initial Number of Features ^a^	Final Number of Features	Number of Imputed Values	Percent Total Values Imputed
Move	87	87	72	42	144	3.94%
Shoot	115	113	72	40	165	3.65%
Communicate	84	84	72	42	136	3.85%
Navigate	83	81	72	46	210	5.64%
Sustain	110	109	72	41	190	4.25%

Note. For Move, 42 features were used to predict 87 observations. For Shoot, 40 features were used to predict 113 observations, etc. ^a^ Value is the product of 12 analytes for each of the six calculated features.

**Table 5:** Overview of outlier means (*M*) and standard deviations (*SD*) as distributed by participant and feature.

Domain	*M* Number of Outliers Per Participant	*SD* Number of Outliers Per Participant	*M* Number of Outliers Per Feature	*SD* Number of Outliers Per Feature
Move	1.885	2.563	3.814	2.462
Shoot	1.611	2.152	4.439	2.460
Communicate	1.786	2.334	3.488	2.293
Navigate	2.099	2.125	3.617	2.786
Sustain	1.706	2.225	4.429	2.370

**Table 6:** Participant sample characteristics (N = 115), including means, standard deviations, and ranges.

Characteristic	Mean	StDev	Range
Age (years)	23.02	3.68	18–35
Education (years)	12.69	1.16	10–16
Military Experience (years)	2.53	1.99	0–10
Body Fat (%)	13.24	5.15	4.2–29.4
Body Mass Index (BMI)	26.51	3.35	18.9–35.9
Anaerobic Capacity (Watts)	2639.5	482.2	1478–3804

**Table 7:** Summary of model performance across domains and algorithms.

Domain	Model	Number with *R*^2^ > 0	Mean RMSE	Mean MAE	Mean Directional Predictive Accuracy
Move	Elastic Net	1	1.023	0.824	48.7%
	PLS-R	17	1.055	0.877	50.2%
	Random Forest	39	0.984	0.803	58.4%
Shoot	Elastic Net	0	1.022	0.838	42.3%
	PLS-R	11	1.103	0.921	48.3%
	Random Forest	2	1.069	0.882	45.9%
Communicate	Elastic Net	21	0.998	0.796	50.5%
	PLS-R	39	0.991	0.782	50.7%
	Random Forest	30	1.005	0.800	56.5%
Navigate	Elastic Net	3	0.997	0.826	43.1%
	PLS-R	10	1.072	0.910	50.5%
	Random Forest	14	1.035	0.857	51.7%
Sustain	Elastic Net	1	0.997	0.752	44.5%
	PLS-R	16	1.018	0.786	49.1%
	Random Forest	9	1.057	0.799	46.6%

Note. For each domain–model combination, 100 iterations of 80/20 train-validation splits were performed. The table reports the number of iterations with nontrivial model fit (*R^2^* > 0), the mean root mean squared error (RMSE), the mean absolute error (MAE), and the mean directional predictive accuracy, which is defined as the average proportion of validation samples for which the predicted sign direction matched the observed sign direction.

## Data Availability

Data were collected on a specialized and sensitive military population. Contact the corresponding author for data inquiries.
